# Pharmacological inhibition of coronavirus replication via modulation of host lipid metabolism

**DOI:** 10.1099/jgv.0.002362

**Published:** 2026-11-10

**Authors:** Maria Soultsioti, Montserrat Bárcena, Martin Giera, Eric J. Snijder

**Affiliations:** 1 Molecular Virology Laboratory, Leiden University Center for Infectious Diseases (LUCID), Leiden University Medical Center, Leiden, Netherlands; 2 Section Electron Microscopy, Department of Cell and Chemical Biology, Leiden, Netherlands, University Medical Center; 3 Center for Proteomics and Metabolomics, Leiden University Medical Center, Leiden, Netherlands

**Keywords:** coronavirus, host-directed antivirals, lipids, membranes, metabolism, small-molecule inhibitors

## Abstract

The emergence of severe acute respiratory syndrome coronavirus 2 (SARS-CoV-2) in 2019 and the subsequent coronavirus disease 2019 pandemic highlighted the urgent need for novel therapeutic approaches. Coronaviruses rely extensively on host cell metabolic pathways to support their propagation, and efficient viral replication requires the extensive remodelling of host cell membranes. This includes the formation of specialized membranous structures known as replication organelles that accommodate viral RNA synthesis in the cytoplasm of the infected cell. Other processes depending on host membranes are viral entry, the acquisition of the coronavirus envelope from intracellular membrane compartments and the post-translational conjugation of lipids to certain viral proteins. The endogenous pathways involved in these pivotal steps of coronavirus replication represent attractive targets for host-directed antiviral therapy, which may also reduce the emergence of resistance that often limits the efficacy of direct-acting antiviral drug treatment. Accordingly, recent studies have identified a range of small-molecule inhibitors that target lipid metabolism pathways and display promising antiviral activity against coronaviruses. In this review, we examine the pharmacological modulation of lipid metabolism with small-molecule inhibitors as an antiviral strategy against SARS-CoV-2 and other coronaviruses and discuss the strengths, limitations and translational challenges associated with these compounds. We further consider how advances in our understanding of such virus–host metabolic interactions may facilitate the development of broadly active host-directed antivirals, contributing to pandemic preparedness against known and newly emerging coronaviruses.

## Introduction

Coronaviruses (CoVs) represent pathogens of significant clinical and veterinary importance. They infect a wide range of birds and mammals, including livestock and domestic species, causing mainly respiratory and intestinal infections. These viruses are enveloped, possess a single-stranded, positive-sense RNA genome (gRNA) and are the best-characterized family within the order *Nidovirales* [1, 2]. Although CoVs have been studied for decades [3], their complex replication cycle gained unprecedented public and scientific attention following the emergence of severe acute respiratory syndrome coronavirus 2 (SARS-CoV-2) and the ensuing global health crisis [4, 5].

The coronavirus disease 2019 (COVID-19) pandemic underscored the importance of interdisciplinary research, with ‘-omics’ technologies and genome-wide association studies (GWASs) accelerating the identification of host factors involved in CoV replication. Notably, GWAS revealed that enzymes and regulators involved in host cell fatty acid biosynthesis, including *de novo* lipogenesis (DNL), and cholesterol biosynthesis are critical for the replication of SARS-CoV-2 and other CoVs [6–8]. These observations are consistent with the well-established dependence of CoVs on host cell membranes for pivotal steps of their replication cycle, including viral entry, RNA synthesis and progeny virion assembly (reviewed in [9], Fig. 1). Briefly, CoV entry is mediated by binding to a host cell receptor [e.g. ACE2 for SARS-CoV-2, DPP4 for Middle East respiratory syndrome coronavirus (MERS-CoV)]. Subsequently, the viral spike (S) protein mediates fusion between the viral envelope and host membranes, a process that can occur either at the plasma membrane or within endosomal compartments [10]. Once released into the cytosol, the positive-strand gRNA is translated into two large replicase polyproteins, pp1a and pp1ab (Fig. 1). These are subsequently processed by multiple viral proteases into the 16 nonstructural proteins (nsps) that drive viral RNA synthesis and a range of virus–host interactions [9, 11]. CoVs induce the extensive remodelling of endoplasmic reticulum (ER) membranes (Fig. 1), resulting in a perinuclear network of membrane structures collectively referred to as the replication organelle (RO) [12]. The CoV RO comprises double-membrane vesicles (DMVs), paired or convoluted membranes and double-membrane spherules (Fig. 1). Among these, the DMVs constitute the most abundant structures [13] and – importantly – they were shown to constitute the primary site of viral RNA synthesis, supporting both genome replication and the transcription of subgenomic mRNAs [13].

**Fig. 1.**
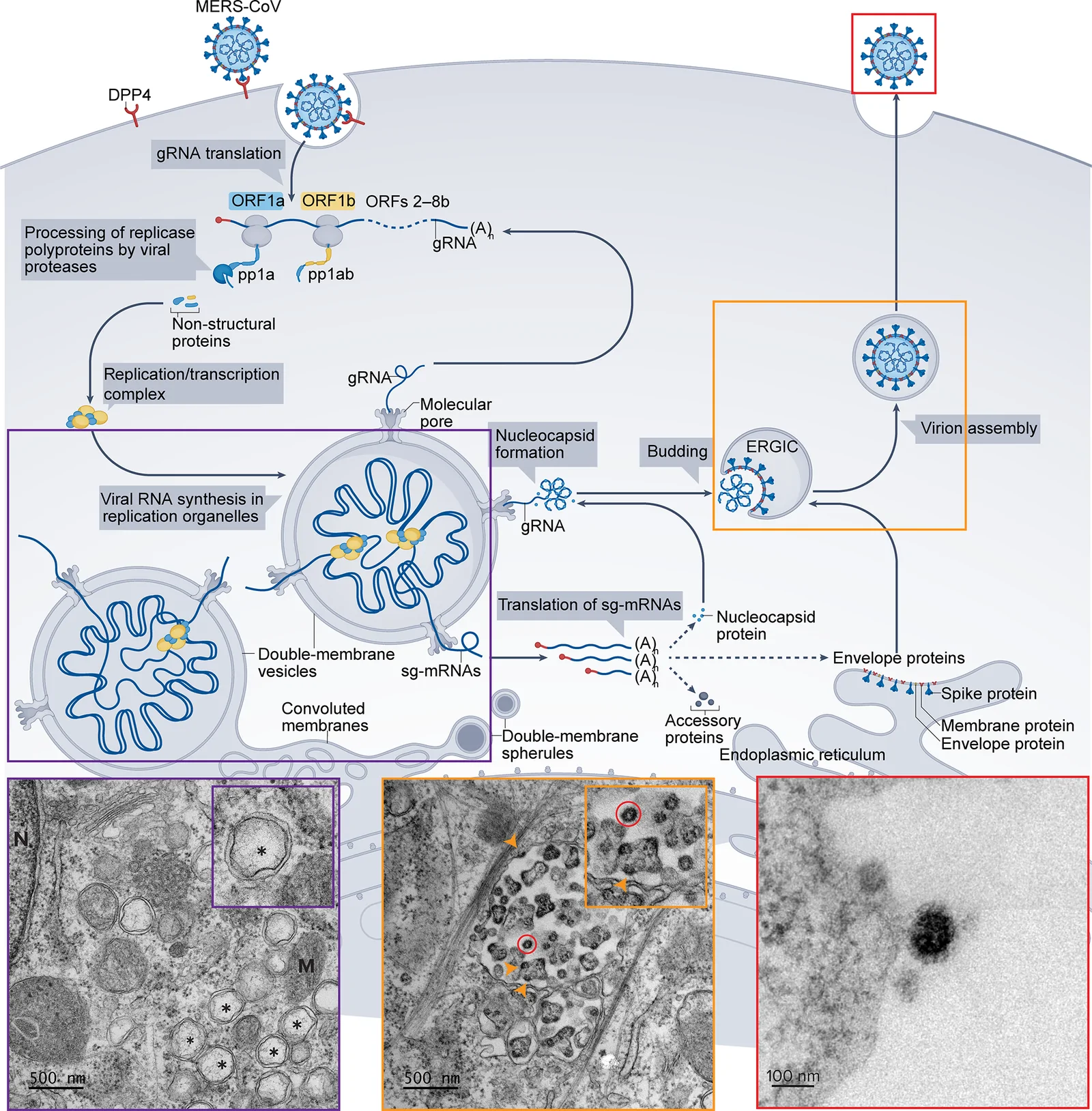
Schematic overview of the CoV replication cycle, using MERS-CoV as an example. The main events taking place during viral replication are illustrated: binding and entry, translation of viral nsps, polyprotein processing, RO formation, replication of gRNA, generation and translation of subgenomic mRNAs, nucleocapsid formation and budding, virus particle assembly and release. The steps boxed in the schematic are visualized below by electron micrographs from MERS-CoV-infected Huh7 cells [64]: a fully assembled extracellular virion (red panel), network of perinuclear ROs (purple panel) and progeny virion assembly and budding (orange panel). In the middle EM image, DMVs are annotated with an asterisk, the nucleus is annotated with N and mitochondria are annotated with M. In the rightmost EM image, examples of budding nucleocapsid structures are annotated with orange arrowheads, and a budded virion is circled in red. Schematic adapted from the SARS-CoV-2 replication cycle in [122] and [11], under the terms of CC-BY 4.0 licence, and modified to depict the MERS-CoV entry and genome organization.

Viral transcripts are thought to be exported from the DMV interior to the cytosol via a molecular pore complex consisting of nsp3 and nsp4 that spans the DMV’s double membrane [14–16]. Once exported, subgenomic mRNAs are translated into structural or accessory proteins. In parallel, newly synthesized gRNAs serve either as templates for further nsp synthesis or associate with nucleocapsid (N) proteins to form nucleocapsids. These acquire a lipid envelope by budding from host membranes along the secretory pathway, where the viral envelope proteins S, membrane (M) and envelope (E) have accumulated. Virus assembly mainly occurs at the ER–Golgi intermediate compartment or Golgi complex [17, 18]. While progeny virion release was historically thought to occur via the conventional biosynthetic secretory pathway [19], recent evidence indicates that CoVs may also exploit lysosomal trafficking routes for their egress from the infected cell [20–23].

Given the need for broad-spectrum antiviral strategies and the central role of host membranes during multiple stages of the CoV replication cycle, increasing attention has focused on targeting host lipid metabolism as an antiviral strategy. While previous reviews [24, 25] have addressed the roles of membranes and specific lipid species in CoV replication, a critical, pharmacologically oriented evaluation of the tools used to interrogate these pathways remains lacking. In this review, we highlight small-molecule inhibitors targeting enzymes within lipid metabolic pathways as emerging starting points for the development of therapeutic interventions against CoVs. We discuss mechanistic insights that underpin their antiviral potential, as well as their strengths, limitations and prospective clinical relevance.

## Pharmacological modulation of host lipid metabolism as an antiviral strategy

### Inhibitors of sterol regulatory element-binding proteins

Key proviral host factors identified in the aforementioned GWAS efforts on CoVs include the sterol regulatory element (SRE)-binding proteins 1 and -2 (SREBP-1 and -2) and their upstream activator, SREBP cleavage-activating protein (SCAP). Together, SCAP and SREBP-1/2 serve as central regulators of DNL and cholesterol metabolism, respectively [26]. Normally, SREBPs reside in the ER as inactive precursor complexes bound to SCAP. When sterol levels are sufficient, the SCAP/SREBP complex is retained in the ER through its interaction with the insulin-inducible gene (Insig) protein [27]. This retention is mediated by cholesterol binding to SCAP’s sterol-sensing domain, which induces a conformational change [28, 29]. In contrast, under low-sterol conditions, the SCAP/SREBP complex is released from Insig and relocates to the Golgi complex, where two sequential cleavage events release the active N-terminal domain of SREBPs. Subsequently, this domain translocates to the nucleus, where it acts as a transcription factor, binding to SRE promoter regions to induce genes involved in lipogenesis (SREBP-1-mediated transcription of *acc*, *fasn* and other genes) and cholesterol biosynthesis (SREBP-2-mediated transcription of *hmgcs* and other genes) (Fig. 2a) [30].

**Fig. 2.**
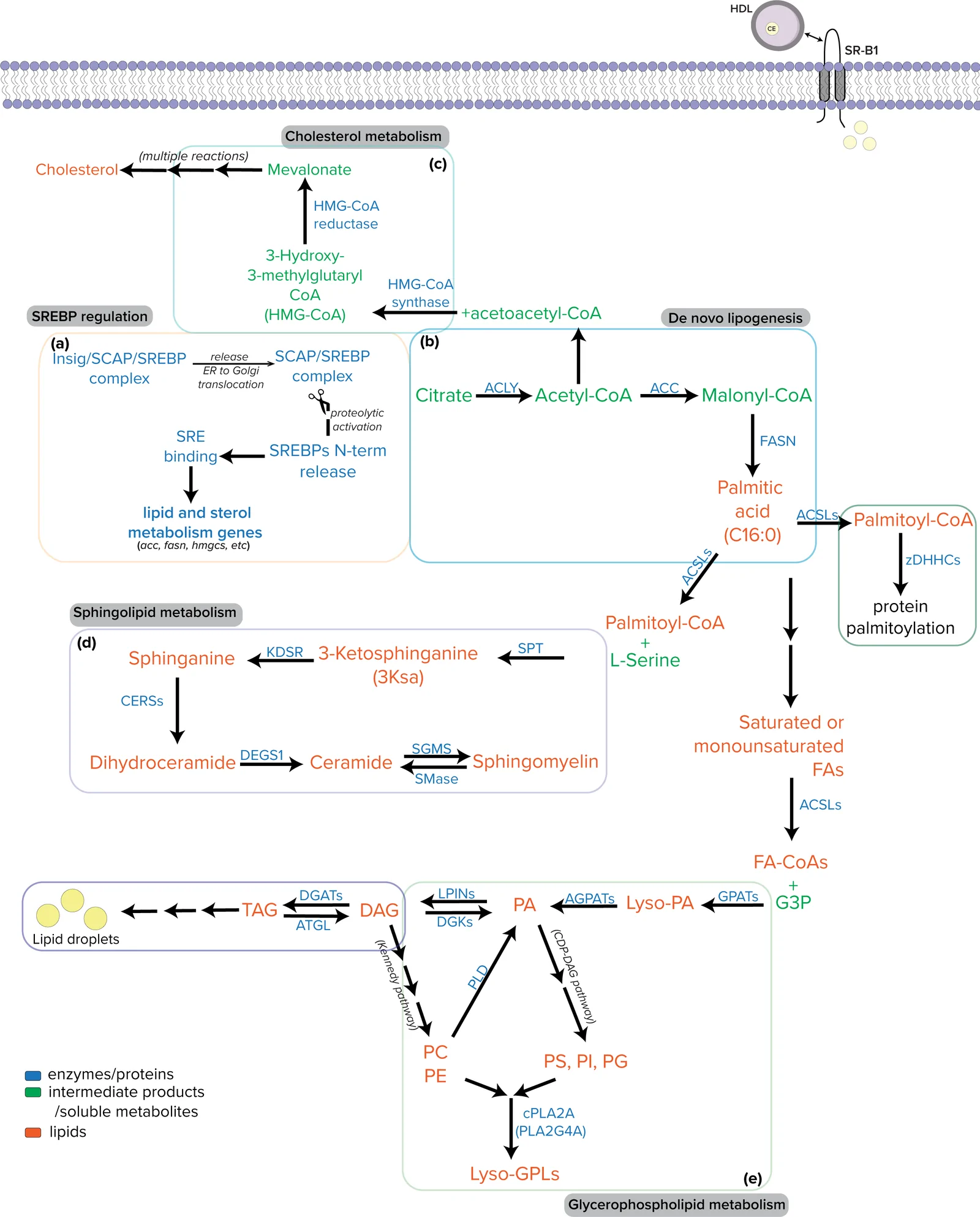
Simplified overview of the host cell lipid metabolism pathways discussed in this review. Regulation of host lipid metabolism via SREBPs is key to the generation of enzymes (a) that drive DNL (b) and cholesterol biosynthesis (c). Fatty acids generated through DNL (a) are used further downstream for protein lipidation, sphingolipid synthesis (d), phospholipid metabolism (e) and the formation of lipid droplets. The cell-surface receptor scavenger receptor class B type 1 facilitates the uptake of high-density lipoprotein (HDL)-bound cholesterol, in the form of cholesteryl esters (CE), from the bloodstream by binding HDL particles and facilitating the transfer of cholesteryl esters from the lipoprotein inside the cell [77, 119, 123, 124]. SCAP, SREBP cleavage-activating protein; SREBPs, sterol regulatory element-binding proteins; SRE, sterol regulatory element promoter region; hmgcs, HMG-CoA synthase; ACLY, ATP citrate lyase; ACC, acetyl-CoA carboxylase; FASN, fatty acid synthase; ACSLs; long-chain fatty acyl-CoA synthetases; zDHHCs, zinc finger DHHC-type palmitoyltransferases; SPT, serine palmitoyltransferase; KDSR, 3-ketodihydrosphingosine reductase; CERs, ceramide synthases; DEGS1, delta 4-desaturase, sphingolipid 1; SGMS, sphingomyelin synthase; SMase, sphingomyelinase; G3P, glycerol-3-phosphate; GPATs, glycerol-3-phosphate O-acyltransferases; PA, phosphatidic acid; AGPATs, 1-acylglycerol-3-phosphate O-acyltransferases; LPINs, lipins; DAG, diacylglycerol; DGKs, DAG kinases; DGATs, diacylglycerol O-acyltransferases; TAG, triacylglycerol; ATGL, adipose triglyceride lipase; PC, phosphatidylcholine; PE, phosphatidylethanolamine; PLD, phospholipase D; PS, phosphatidylserine; PI, phosphatidylinositol; PG, phosphatidylglycerol; cPLA2A, cytosolic phospholipase A; lyso-GPLs, lyso(glycero)phospholipids.

In 2019 and preceding the pandemic GWAS efforts, a synthetic retinoic acid receptor alpha (RARα) agonist, AM580, was reported to have potent antiviral properties against the highly pathogenic MERS-CoV [31]. Interestingly, AM580 inhibited MERS-CoV replication *in vitro* in multiple cell lines, as well as *in vivo* in a mouse model. Moreover, upon AM580 treatment, DMV structures were lacking in MERS-CoV-infected cells, leading the authors to conclude that its mechanism of action involved RO formation. According to the same study, while AM580 was initially designed to bind RAR*α*, it could also bind to SREBPs and more potently to SREBP-1. This finding suggested that DNL pathways and lipid metabolism are crucial for DMV formation in CoV-infected cells. It is noteworthy that, while computational modelling suggested an interaction between AM580 and SREBPs, significant functional effects in biochemical assays were only observed when using high-micromolar concentrations of the compound (25–100 µM) [31]. In comparison, AM580 displays sub-nanomolar potency toward its original target, RAR*α*, with a half-maximal effective concentration (EC_50_ value) of 0.36 nM [32].

To further confirm a general proviral role for SREBPs during MERS-CoV replication, Yuan *et al*. [31] also tested betulin and PF-429242 for antiviral activity. Betulin, a pentacyclic triterpene extracted from birch bark, binds to SCAP, stimulating the interaction between Insig and SCAP and thus preventing the translocation of the SCAP/SREBP complex to the Golgi compartment and SREBP’s subsequent activation [33]. PF-429242 is a competitive, reversible aminopyrrolidine amide-based inhibitor of the site-1 protease [34], one of the two enzymes responsible for the cleavages that activate the N-terminal domain of SREBPs. Both small-molecule inhibitors exhibited anti-MERS-CoV activity in a dose-response manner in a cytopathic effect reduction assay, strengthening the author’s conclusions regarding the SREBP dependence of MERS-CoV replication.

Subsequent studies on SARS-CoV-2 in cell culture-based infection models explored the potential antiviral effects of AM580 against this newly emerged CoV [35–37]. These studies reported potent antiviral properties against SARS-CoV-2 and suggested that SREBP-1 was an interesting druggable target. However, comparative analyses by Soares and colleagues including two established SREBP pathway inhibitors, fatostatin and betulin, yielded mixed results [37]. Like betulin (see above), fatostatin, a diarylthiazole compound, prevents ER-to-Golgi transport of the SCAP/SREBP complex by binding directly to SCAP, albeit using a different binding site outside the Insig-binding domain [38, 39]. According to Soares *et al*., betulin did not exert any antiviral effect against SARS-CoV-2, while AM580 and fatostatin prevented viral replication in cell culture [37]. Moreover, the authors observed that the inhibitory effect of fatostatin against SARS-CoV-2 was more prominent than AM580’s antiviral effect. Interestingly, a separate study also made use of betulin and fatostatin, independently of studying AM580 [40], as part of an *in vitro* screening of inhibitors that affect lipid metabolism pathways. Notably, in this study, neither betulin nor fatostatin exhibited an antiviral effect. It is worth noting that Chu *et al*. [40] used human embryonic kidney (HEK293T) cells expressing the ACE2 receptor, while Soares *et al*. employed the more physiologically relevant Calu3 lung cells [37], a technical difference that may have a major impact in host factor studies. Overall, while several findings, either with the use of small-molecule inhibitors or gene silencing approaches [31, 36, 37], have supported a role for SREBPs in CoV replication, results across laboratories remain partly inconsistent or inconclusive.

#### Inhibitors of acetyl-CoA carboxylase and fatty acid synthase

Acetyl-CoA carboxylase (ACC) and fatty acid synthase (FASN) are critical enzymes in the DNL pathway, whose activity ultimately leads to the generation of fatty acids. ACC catalyses the carboxylation of acetyl-CoA to generate malonyl-CoA (Fig. 2b) and is negatively regulated by AMP-activated protein kinase (AMPK), which phosphorylates and (reversibly) inactivates ACC under low-energy conditions [41]. The role of ACC has been studied in the context of various diseases, such as cancer, obesity and diabetes (reviewed in [41]), but it has also gained attention in relation to viral infections and how viruses try to take advantage of this branch of the DNL pathway. In the context of SARS-CoV-2 research, Williams *et al*. reported that inhibition of ACC with the synthetic compound 5-(tetradecyloxy)-2-furoic acid (TOFA) potently inhibited viral replication, especially when compared to other small-molecule inhibitors targeting enzymes of the DNL or fatty acid oxidation pathways [42]. Immunofluorescence microscopy revealed a reduction in the number of puncta positive for dsRNA, a marker for intracellular viral RNA synthesis, indicating that ACC inhibition blocks an early step in the viral replication cycle.

Following the conversion of acetyl-CoA into malonyl-CoA, the latter serves as a key substrate for FASN, a large multi-domain enzyme that catalyses a series of reactions that lead to the generation of palmitic acid (C16 : 0) [43] (Fig. 2b). Similar to ACC, FASN has been implicated in the replication cycle of multiple viral pathogens that utilize the end products of fatty acid synthesis to support their replication. These fatty acids can serve several functions, including acting as precursors for the synthesis of other lipids (such as phospholipids), as an energy source for *β*-oxidation, or as substrates for post-translational modifications such as palmitoylation (for a recent, comprehensive review on the role of FASN in viral replication, we direct the reader to [44]).

A study by Chu and colleagues was among the first to explore the potential of FASN inhibition as a strategy to limit SARS-CoV-2 replication [40]. A short-hairpin RNA library was used to identify potential proviral host factors, with ACC and FASN emerging as top candidates. The authors then proceeded to screen ten FASN inhibitors alongside inhibitors of other host factors, such as the SCAP inhibitors betulin and fatostatin (discussed above). Most of these FASN inhibitors showed antiviral activity *in vitro,* with C75, Orlistat, TVB-2640 and its analogue TVB-3166 (Fig. 3) being among the most promising candidates. Subsequently, the efficacy of Orlistat and TVB-2640 was investigated *in vivo*, given their status in ongoing clinical trials and established safety profiles. Notably, Orlistat, an FDA-approved anti-obesity drug that inhibits both gastric lipases and FASN [45], emerged as a potential antiviral compound from two independent SARS-CoV-2 studies [42, 46], further confirming the importance of this branch of lipid metabolism in CoV replication. Mechanistic insights from these studies mainly pointed to disrupted triacylglycerol (TAG) storage in lipid droplets. However, Williams and colleagues also reported that palmitoylation of the viral S and E proteins was impaired based on FASN knockout experiments in which addition of palmitic acid could partially rescue the observed defect [42]. Moreover, in a separate study, TVB-3166 was shown to inhibit both SARS-CoV-2 and the human coronavirus 229E (HCoV-229E), *in vitro* and *in vivo* [47]. Further biochemical analysis revealed reduced S protein palmitoylation, consistent with FASN inhibition and reduced palmitate availability.

**Fig. 3.**
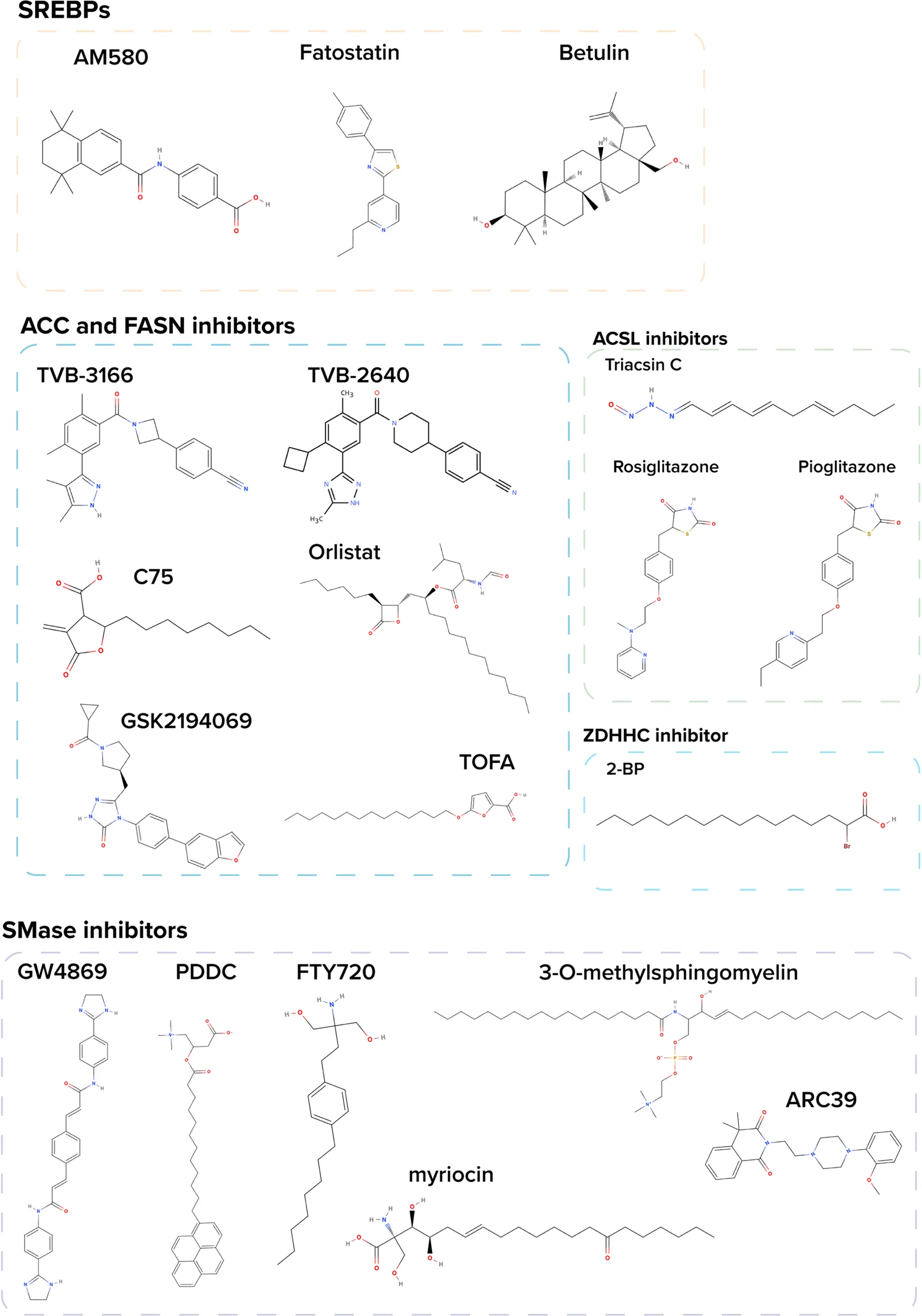

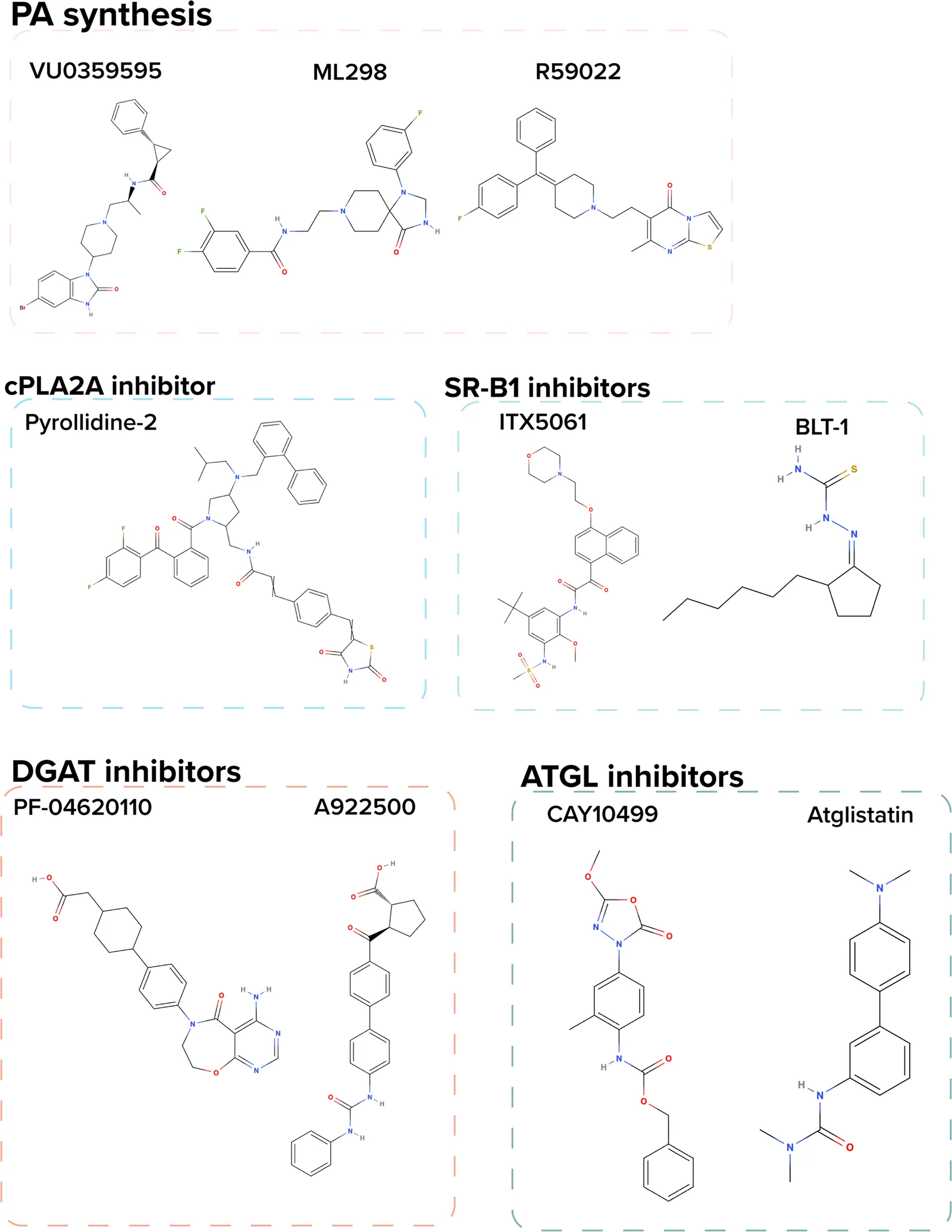
Overview of publicly available chemical structures of the small-molecule inhibitors described in this review, categorized per target [125].

Palmitoylation is catalysed by the zinc finger DHHC-type (zDHHC) family of palmitoyltransferases [48], comprising 23 isoforms. At the moment, isoform-specific zDHHC inhibitors are not available, and the most commonly used universal palmitoylation inhibitor is 2-bromopalmitate (2-BP), a straight-chain, non-metabolizable fatty acid (palmitate) analogue, which is believed to act by covalently modifying zDHHC enzymes, thus preventing binding of palmitate and its transfer to target proteins [49]. However, 2-BP was originally discovered as a broad inhibitor of membrane-bound lipid metabolism enzymes [50], and later studies indeed confirmed its promiscuous activity on various lipid metabolism-related enzymes, essentially highlighting the need for careful data interpretation when 2-BP is used in palmitoylation studies [51, 52].

Palmitoylation is known to be crucial for the CoV S and E proteins, as it promotes their proper membrane association, stability and function in viral assembly and infectivity [53–61]. Against this background, it is not surprising that several recent studies have identified palmitoyltransferase enzymes as potential antiviral targets via loss-of-function approaches [60, 62], while indirect inhibition of palmitoylation has also shown efficacy against CoVs, including SARS-CoV-2. More specifically, three independent studies showcased the antiviral effect of 2-BP treatment [42, 62, 63], consistent with earlier findings in the murine CoV MHV-A59 model [53]. Ramadan *et al*. identified two specific zDHHC9 inhibitors, compounds 13 and 25, which exhibited anti-SARS-CoV-2 activity, albeit with lower potency than the non-specific palmitoylation inhibitor 2-BP [62].

A recent MERS-CoV study essentially integrated the concepts described above by directly comparing the effects of TOFA, TVB-2640 and 2-BP [64]. Interestingly, inhibition of DNL using TOFA and TVB-2640 did not affect viral RNA synthesis but instead impaired a later stage of the replication cycle. Electron microscopy revealed that TOFA treatment of MERS-CoV-infected cells had no effect on DMV formation yet markedly impaired the assembly and release of viral progeny. Biochemical analyses further indicated that trafficking and post-translational modification of viral envelope proteins was impaired, while palmitoyltransferase inhibition with 2-BP recapitulated the effects observed upon TOFA treatment. Supplementation of TOFA-treated infected cells with exogenous palmitic acid partially rescued these defects [64]. Collectively, these findings highlight that S and E protein palmitoylation is required for efficient MERS-CoV assembly and release.

### Inhibitors of serine palmitoyltransferase and sphingomyelinases

Sphingolipids are a diverse class of lipids that is characterized by a sphingosine backbone. They are derived from ceramide (Fig. 2d) and have a plethora of biological functions, both as structural membrane components and as secondary messenger molecules [65, 66]. Together with cholesterol and glycosylphosphatidylinositol-anchored proteins, sphingolipids form complexes that organize into highly dynamic membrane nanodomains known as lipid rafts, which concentrate specific lipids and proteins within the membrane [67].

CoVs have long been postulated to exploit lipid rafts, particularly at the plasma membrane, to enhance viral entry and syncytium formation [68–70]. A study by Mesquita and colleagues on the palmitoylation of the SARS-CoV-2 S protein revealed that the viral envelope has an unusual lipid composition, probably reflecting the membrane composition at the viral budding site [48]. Interestingly, the viral envelope was relatively poor in phospholipids and enriched in phosphatidic acid (PA) as well as various species of sphingolipids. Combined with their data on S protein palmitoylation, the authors concluded that palmitoylated SARS-CoV-2 S protein has an affinity for lipid rafts, probably already during the virion budding stage. In the same study, two broad inhibitors of *de novo* sphingolipid synthesis, myriocin, a fungal sphingoid non-proteinogenic *α*-amino acid, and its synthetic analogue FTY720 (Fig. 3), were employed to assess the importance of this lipid class for SARS-CoV-2 replication, with both exhibiting potent antiviral activity.

Notably, a recent study by Salisch *et al*. pursued a different line of investigation, following up on the results of a global sphingolipid profiling in cells infected with HCoV-229E, SARS-CoV-2 and MERS-CoV [71]. Infection with all three viruses resulted in the time-dependent deregulation of sphingolipid homeostasis, characterized by a global decrease of sphingomyelin (SM) lipid species and a simultaneous increase of total ceramide levels, consistent with enhanced SM hydrolysis to ceramide by pH-dependent, cellular sphingomyelinases (SMases). Subsequently, in order to investigate the functional consequences of this deregulation, the authors employed small-molecule inhibitors and gene silencing to modulate the activity of acid and neutral SMases (aSMases and nSMases, respectively). For the pharmacological inhibition of aSMases, the authors made use of two aSMase-specific inhibitors, 1-aminodecylidene bis-phosphonic acid (ARC39) [72] and PCK310 [73], while for the inhibition of nSMases, they employed 3-O-methyl-sphingomyelin [74], GW4869 [75] and the nSMase2-specific inhibitor PDDC [76]. Inhibition of aSMases with either compound resulted in SARS-CoV-2-specific inhibition, as antiviral activity against HCoV-229E or MERS-CoV was not observed. In contrast, inhibition of nSMases exhibited broad antiviral activity against all three CoVs tested, pointing to a conserved, CoV-wide role for nSMases.

### Inhibitors of PA synthesis

Although structurally PA is the simplest phospholipid, its unique biophysical properties turn it into a key lipid metabolite. PA has many functions as a cellular signalling molecule in the response to stimuli, but it influences membrane structure and dynamics, as well as protein–lipid interactions [77]. A recent study by Tabata *et al*. revealed that enzymes catalysing the biosynthesis of PA, acylglycerolphosphate acyltransferase (AGPAT) 1 and 2 (Fig. 2e), are enriched in the membrane bilayer of DMVs induced by hepatitis C virus (HCV) [78]. In view of the similarities between these DMVs and CoV-induced DMVs, the authors investigated whether AGPATs and alternative PA-synthesizing enzymes are important for SARS-CoV-2 DMV formation. While the authors primarily used loss-of-function approaches to study the roles of AGPATs, they employed the pharmacological inhibitors ML298, VU0359595 and R59022 to probe other PA-synthesizing pathways. More specifically, ML298 is a specific inhibitor of phospholipase 2 (PLD2) [79], VU0359595 is PLD1-specific [80, 81] and R59022 is a broad-acting diacylglycerol kinase inhibitor [82]. All three compounds inhibited SARS-CoV-2 replication, albeit with moderate efficacy (~50% inhibition in the assays used), whereas a more pronounced effect was observed when inhibitors targeting different enzymes were combined. To directly assess the impact on DMV biogenesis, the authors employed a viral replication-independent system in which co-expression of SARS-CoV-2 nsp3 and nsp4 induces transformation of ER membranes into DMVs that resemble the membrane structures formed during viral replication [15]. Upon combining this system with small-molecule inhibitors of PA synthesis and genetic depletion of AGPATs, Tabata *et al*. concluded that mechanistically, the inhibition of PA synthesis impairs DMV formation, possibly due to disruption of the membrane fusion and fission events required for CoV RO formation [83].

### Inhibitors of cytosolic phospholipase A

Phospholipases comprise a large group of membrane-associated enzymes that catalyse the hydrolysis of acyl and phosphate ester bonds in phospholipids, resulting in the release of a fatty acid and the formation of lysophospholipids (Fig. 2e) [84]. Phospholipases are classified according to the position of the bond they hydrolyse in the phospholipid backbone. According to this nomenclature, five major classes of mammalian phospholipases are recognized: phospholipase A_1_ (PLA_1_), A_2_ (PLA_2_), B (PLB), C (PLC) and D (PLD) [85]. Among these, PLA_2_ enzymes are the most extensively characterized. Enzymes belonging to this class are further subdivided into six functionally and structurally distinct subgroups: cytosolic PLA_2_ (cPLA_2_, PLA2G4A), secreted PLA_2_ (sPLA_2_), calcium-independent PLA_2_ (iPLA_2_), lysosomal PLA_2_ (LPLA_2_), platelet-activating factor acetylhydrolases (PAF-AH) and adipose-specific PLA_2_ (AdPLA). Within the cPLA_2_ (PLA2G4) group, six isoforms have been identified: cPLA_2_*α*, -*β*, -*γ*, -*δ*, -*ε* and -*ζ*. Among these, cPLA_2_*α* is the best characterized, while little is known about the *in vivo* functions of the remaining isoforms [85, 86].

Inhibition of cPLA_2_*α* by pyrollidine-2 (py-2) was reported to impair HCV and dengue virus replication by blocking the production of infectious progeny [87]. Given that CoVs are also enveloped, positive-sense RNA viruses, their sensitivity to cPLA_2_*α* inhibition was subsequently examined. Interestingly, using HCoV-229E and MERS-CoV infection models, py-2 treatment inhibited a step preceding virion assembly, preventing DMV formation across multiple cell lines [88]. Specifically, cPLA_2_*α*’s activity appeared to be crucial during the early stages of DMV formation, while its role was less critical once DMV formation had been initiated. This early requirement may reflect a need for lysophospholipid production to facilitate the induction of positive membrane curvature during the initial steps of DMV formation.

### Inhibitors of long-chain fatty acyl-CoA synthetases

Long-chain fatty acyl-CoA synthetase (ACSL) enzymes constitute a family of five isoforms (ACSL-1, -3, -4, -5 and -6) whose main function is to activate otherwise inert fatty acids by converting them into fatty acyl-CoA (Fig. 2). This activation serves as a preparatory step for mitochondrial *β*-oxidation and energy production, as well as for incorporation of fatty acids in other biological processes, such as lipid synthesis [89]. While ACSL enzymes have been explored as antiviral targets for other RNA viruses, such as HCV [90, 91] and rotaviruses [92, 93], primarily using the fungal ACSL inhibitor triacsin C (Fig. 3), their involvement in CoV replication had not been explored prior to the COVID-19 pandemic.

During a screen for inhibitors of class III phosphoinositide 3-kinase and fatty acid metabolism, triacsin C emerged as a small-molecule inhibitor of SARS-CoV-2 replication, effective at substantially lower concentrations than other compounds tested in parallel [42]. A second study revealed that inhibition of ACSL4 by rosiglitazone and pioglitazone (Fig. 3), FDA-approved drugs belonging to the thiazolidinedione family that are commonly used for the treatment of type-2 diabetes, also inhibits replication of SARS-CoV-2, HCoV-229E and HCoV-OC43 [94]. However, no further mechanistic insights were obtained from these studies regarding the role of ACSL enzymes in CoV replication.

### Inhibitors of diacylglycerol O-acyltransferases

Diacylglycerol O-acyltransferases (DGATs) 1 and 2 catalyse the esterification of diacylglycerols for the synthesis of TAGs (end of Fig. 2e). The latter represent an important class of neutral lipids, which are synthesized in the ER and channelled into lipid droplets for further storage [95]. Interestingly, multiple independent studies have explored DGAT inhibition as an antiviral strategy against SARS-CoV-2. Most of these utilized the small-molecule inhibitor A922500 [42, 96, 97], while one study used PF-04620110 [46]. Notably, both A922500 [98] and PF-04620110 [99] are selective DGAT1 inhibitors with IC_50_ values in the nanomolar range. While most studies primarily reported a general viral load reduction without offering mechanistic insights, a study by Ricciardi and colleagues attempted to shed light on the connections between DGAT inhibition, lipid droplet biogenesis and SARS-CoV-2 replication [96]. Using a combination of biochemical assays and microscopy, the authors identified a specific region within nsp6, the third viral multi-pass transmembrane protein potentially involved in CoV RO generation, that may mediate interactions between DMVs and lipid droplets. The authors proposed that this region could facilitate the transfer of lipid droplet-derived lipids to the DMVs, possibly supporting DMV expansion.

### Inhibitors of adipose triglyceride lipase

Approximately two decades ago, adipose triglyceride lipase (ATGL) was discovered as the rate-limiting enzyme responsible for lipolysis, driving the hydrolysis of triglycerides (Fig. 2e) and the release of free fatty acids for energy production [100]. Since then, ATGL has been established as a key lipolytic enzyme and crucial regulator of whole-body energy homeostasis [101].

Given ATGL’s essential role in lipid metabolism and building on findings that SARS-CoV-2 replication affects lipid metabolism and lipid droplet homeostasis, Farley *et al*. investigated the contribution of lipolytic pathways to viral replication using Atglistatin, Orlistat and CAY10499 [46]. All three compounds reduced SARS-CoV-2 progeny titres, supporting a proviral role for this branch of lipid catabolism and possibly reflecting a need for energy supplied via this pathway during viral replication. However, only Orlistat and CAY10499 caused a significant reduction in viral titres, whereas Atglistatin treatment resulted in only partial inhibition. The authors interpreted the differential antiviral efficacy as being inversely related to compound specificity, with ATGL-specific inhibitors like Atglistatin exhibiting reduced antiviral activity. In contrast, Orlistat [45, 102] and CAY10499 [103] are relatively non-specific lipase inhibitors [104], which may account for their stronger antiviral activity. However, an alternative explanation for the limited impact of Atglistatin treatment is its species specificity [105], as the compound is active against murine ATGL, but does not inhibit the human enzyme [104, 106]. This limitation has prompted the recent development of NG-497, a selective small-molecule inhibitor of human ATGL [107], which should enable more precise investigation on ATGL function during CoV replication in future studies.

### Inhibitors of scavenger receptor B1

Scavenger receptor class B type 1 (SR-B1) is a cell-surface receptor that mediates the uptake of high-density lipoprotein (HDL)-derived cholesteryl esters (Fig. 2). Upon uptake, cholesteryl esters may either remain intact and be stored in lipid droplets or undergo hydrolysis to generate free cholesterol for membrane biosynthesis and production of hormones and bile acids [108]. Two independent studies reported a consistent anti-SARS-CoV-2 effect upon pharmacological inhibition of SR-B1 [109, 110] using the small-molecule inhibitors ITX 5061 [111] and BLT-1 [112]. A 2020 study by Wei *et al*. focused on the antiviral effect of ITX 5061 and suggested that HDL and SR-B1 mediate binding of S protein to cholesterol-rich plasma membrane domains prior to viral entry. In contrast, McConnel *et al*. concluded that inhibition of viral entry by both small-molecule inhibitors was independent of S protein’s binding on cholesterol-rich domains. Instead, this study pointed to inhibition of endolysosomal acidification, leading to the ‘trapping’ of viral particles within endolysosomal compartments.

It should be noted that Wei and colleagues primarily used Huh-7 cells for their inhibitor studies, whereas McConnel *et al*. employed A549 cells engineered to express the ACE2 receptor. These cell lines differ markedly in their expression of the cell surface protease TMPRSS2 [113, 114], a key determinant of the SARS-CoV-2 entry route. SARS-CoV-2 entry involves receptor binding followed by fusion of the viral envelope with host membranes, a process that can occur either at the plasma membrane or within endosomal compartments [10]. Activation of the fusion activity of the CoV S protein requires sequential proteolytic cleavages at the S1/S2 and S2’ sites. The S1/S2 site is cleaved by furin proteases during virion biogenesis, whereas S2’ cleavage is mediated by TMPRSS2 at the cell surface, promoting direct fusion at the plasma membrane. In cells with low TMPRSS2 levels, the virus particle is instead internalized via endocytosis, and S2′ cleavage is carried out by endosomal cathepsins. This enables fusion of viral envelope with endosomal membranes and the subsequent release of the nucleocapsid into the cytosol. Accordingly, the use of unmodified A549 cells likely biases entry toward the endosomal pathway as these cells generally express negligible levels of TMPRSS2 [113, 114]. Overall, while both studies reported that pharmacological inhibition and subsequent gene silencing of SR-B1 block SARS-CoV-2 entry, the underlying mechanisms are likely influenced by differences in the experimental design and cellular models used.

## Summary and translational perspectives

Traditionally, most FDA-approved antivirals have been designed to directly target viral enzymes and disrupt their activity. However, this approach is frequently undermined by the rapid emergence of drug-resistant viruses [115, 116], highlighting the need for alternative antiviral approaches. In addition to the development of combination therapies and potentially partly overlapping with this strategy, an increasingly explored path is the use of host-directed antivirals that target cellular factors essential for viral replication. The rationale behind this approach lies in its potential to reduce the likelihood of resistance emergence, given that host genetic variability is considerably lower than that of viruses. Moreover, host-directed antivirals offer the possibility of drug design for the intentional targeting of multiple pathways or host factors (polypharmacology) [117]. Currently, most FDA-approved host-directed antivirals are interferon-based and act by stimulating the innate immune response. In parallel, numerous host targets and cellular pathways, including kinases, heat shock proteins, nucleotide metabolism pathways, the ubiquitin-proteasome system, ion channels and transporters, are currently being explored for their suitability as therapeutic targets in antiviral drug development [118].

As summarized in this review, preclinical studies have identified a diverse range of lipid metabolism inhibitors with distinct chemical structures (Fig. 3) and molecular targets. These compounds show considerable promise for further development into clinically relevant anti-CoV agents (Table 1). However, the vast majority of these studies focused on SARS-CoV-2, leaving unresolved questions regarding the broader applicability of these findings to other CoVs and the extent to which they reflect evolutionarily conserved mechanisms. Moreover, many of the studies discussed in this review relied on multi-cycle, cytopathic effect-based assays performed at low multiplicities of infection. Under these conditions, viruses undergo multiple rounds of replication over several days, making it difficult to attribute the observed antiviral effects to specific stages of the viral replication cycle or to define the precise roles of individual host factors. Consequently, important mechanistic questions remain unanswered.

**Table 1.** Overview of small molecules targeting lipid metabolism-associated enzymes and their impact on CoV replication

Target	Compound	Virus	IC_50_/CC_50_ (µM) (cell model)	Impact on CoV replication cycle^*^		Pharmaceutical type/class	Reference
SREBPs	AM580	MERS-CoV	0.25/118 (Huh7)	(+)	Absence of DMVs	Synthetic compound	[31]
	Betulin PF-429242	MERS-CoV	n/a	(+)	Unknown	Birch extract (natural product) synthetic compound	[31]
	AM580	SARS-CoV-2	7.6/126 (VeroE6)	(+)	Unknown	Synthetic compound	[35]
	AM580	SARS-CoV-2	n/a	(+)	Unknown	Synthetic compound	[36]
	Fatostatin AM580 Betulin	SARS-CoV-2 SARS-CoV-2 SARS-CoV-2	14.15/- n/a n/a (Calu3)	(+) (±) (−)	Unknown	Synthetic compound natural product	[37]
	Fatostatin Betulin	SARS-CoV-2	n/a	(−)	Unknown	Synthetic compound natural product	[40]
ACC	TOFA	SARS-CoV-2	1.4/>100 (Calu3)	(+)	Reduced viral titres and ROs	Synthetic compound	[42]
	TOFA	MERS-CoV	n/a	(+)	Reduced viral titres/assembly defects	Synthetic compound	[64]
FASN	Orlistat C75	SARS-CoV-2	21.25/>1000 220.8/>50 (Calu3)	(+)	Reduced viral titres/palmitoylation?	Synthetic compounds	[42]
	TVB3664 TVB2640 TVB3166 Orlistat GSK-2194069 FASN-IN-4 FT113	SARS-CoV-2	0.00007 0.004 0.011 0.4 0.0009 0.019 0.017 (HEK293T-hACE2)	(+)	Unknown	Synthetic compounds	[40]
	GSK-2194069 Orlistat	SARS-CoV-2	0.0019 0.094 (HEK293T-hACE2)	(+)	Reduced viral titres/unknown	Synthetic compounds	[46]
	TVB-3166	SARS-CoV-2 HCoV-229E	n/a	(+) (+)	Inhibition of S protein palmitoylation	Synthetic compound	[47]
Protein lipidation (palmitoylation)	2-BP	SARS-CoV-2	23/>200 (Calu3)	(+)	Reduced viral titres/palmitoylation?	Straight-chain modified fatty acid	[42]
	2-BP Compound 13 Compound 25	SARS-CoV-2	n/a	(+)	Inhibition of S protein palmitoylation	Straight-chain modified fatty acid synthetic compound Synthetic compound	[62]
	2-BP	HCoV-229E HCoV-OC43 MHV-A59	n/a	(+) (+) (+)	Reduced viral titres/unknown	Straight-chain modified fatty acid	[63]
Sphingolipid synthesis	Myriocin FTY720	SARS-CoV-2	n/a	(+)	Unknown	Fungal-derived sphingoid synthetic analogue	[60]
aSMases	ARC39 PCK310	SARS-CoV-2 MERS-CoV HCoV-229E	n/a	(+) (−) (−)	Reduced viral titres	Synthetic compounds	[71]
nSMases	3-O-Methyl-sphingomyelin GW4869 PDDC	SARS-CoV-2 MERS-CoV HCoV-229E	n/a	(+) (+) (+)	Reduced viral titres and ROs	SM analogue synthetic compound synthetic compound	[71]
PA synthesis	ML298 VU0359595 R59022	SARS-CoV-2	42/- 17/- 28/- (Calu3 cells)	(+)	DMV formation	Synthetic compounds	[78]
cPLA2A	Pyrrolidine-2	HCoV-229E MERS-CoV	n/a	(+) (+)	Reduced viral titres and ROs	Synthetic compound	[88]
ACSL	Triacsin C	SARS-CoV-2	0.04/>50 (Calu3)	(+)	Post-entry (?)	Fungal derived (natural product)	[42]
	Rosiglitazone Pioglitazone	SARS-CoV-2 HCoV-229E HCoV-OC43	n/a 7–12/>100 5–10/ >100 (LLC-MK2, VeroE6)	(+) (+) (+)	Unknown	Synthetic compounds	[94]
DGAT	A922500	SARS-CoV-2	4/>50 (Calu3)	(+)	Reduced viral titres	Synthetic compound	[42]
	PF-04620110	SARS-CoV-2	0.49/ - (HEK293T-hACE2)	(+)	Reduced viral titres/unknown	Synthetic compound	[46]
	A922500	SARS-CoV-2	n/a	(+)	Reduced viral titres	Synthetic compound	[96]
	A922500	SARS-CoV-2	3.8 (VeroE6)	(+)	Reduced viral titres	Synthetic compound	[97]
ATGL	Atglistatin Orlistat CAY10499	SARS-CoV-2 SARS-CoV-2 SARS-CoV-2	>10 00.01 0.16 (HEK293T-hACE2)	(±) (+) (+)	Reduced viral titres/unknown	Synthetic compounds	[46]
SR-B1	ITX 5061 BLT-1	SARS-CoV-2	22.5/>300 180.8/113 (A549-ACE2)	(+)	Entry	Synthetic compounds	[109, 110]

* (+) inhibition, (−) lack of inhibition and (±) weak inhibitory effect (if indicated as such by the original study). Unknown indicates the use of a multi-cycle experimental setup, and conclusions on the step of viral cycle affected cannot be drawn. n/a: IC_50_ and CC_50_ not reported in this study.

Another important limitation is the widespread use of established cell-line models, which may not accurately recapitulate the natural physiological environment of the primary target cells infected by SARS-CoV-2 and other CoVs, such as those in the respiratory tract. Although numerous studies have implicated host lipid metabolism factors in CoV replication, definitive conclusions regarding the extent to which SARS-CoV-2, MERS-CoV and potentially other CoVs depend on these pathways – and whether important virus-specific differences exist – will require systematic evaluation. Ideally, such studies should be conducted using standardized experimental infection models, both *in vitro* and *in vivo*, to enable robust and meaningful comparisons across CoVs.

To assess our conclusions more quantitatively, we revisited the studies included in Table 1 and extracted the reported antiviral (IC_50_) and cytotoxicity (CC_50_) values for each small-molecule inhibitor. Across the cited studies, only 30 of 52 compound-study evaluations (~58%) reported IC_50_ values, while CC_50_ values were available for only 12 evaluations (~23%). Moreover, despite the limited dataset, the reported IC_50_ values were generally in the micromolar range, with only one exception, and the corresponding selectivity indices were relatively modest. In our view, the available data do not allow for a rigorous assessment of the antiviral potential of these inhibitors in a clinically relevant setting. Therefore, in this review, we primarily evaluated these compounds based on their effects on the targeted pathways rather than their antiviral potency alone. To enable a more comprehensive assessment of antiviral efficacy, a side-by-side comparison of these small-molecule inhibitors using a relevant cell model and standardized experimental assays would be required.

Despite considerable interest in lipid metabolism as a therapeutic target for a range of diseases, the development of small-molecule inhibitors targeting these pathways has historically proven challenging [119]. In particular, achieving sufficient therapeutic efficacy while maintaining an acceptable safety profile remains a major hurdle. As host-directed antivirals target cellular pathways rather than viral components, some degree of toxicity or adverse effects is generally anticipated. In certain clinical contexts, such as cancer therapy, these side effects may be considered acceptable and may even reflect the intended mechanism of action, provided that the therapeutic benefits substantially outweigh the associated risks [120]. In the context of antiviral therapy, however, tolerance for treatment-associated toxicity may be lower, particularly for viral infections causing mild or self-limiting disease. Consequently, the clinical utility of host-directed antivirals – and lipid metabolism inhibitors in particular – may depend strongly on disease severity, potentially making such approaches more suitable for highly pathogenic viral infections such as Ebola virus disease or Nipah viral infection. One possible strategy to mitigate toxicity could involve the use of lipid metabolism inhibitors in combination with direct-acting antivirals, thereby enabling shorter treatment durations and/or lower dosing regimens. Nevertheless, for acute respiratory viral infections, where treatment windows are narrow and the target population is large, achieving the therapeutic index required for the successful implementation of host-directed antivirals may prove particularly challenging.

Beyond their direct therapeutic applications, lipid metabolism inhibitors may also serve as valuable tools for uncovering host dependencies and dissecting virus–host interactions. This concept was recently illustrated by a study identifying SM synthase as an essential host factor for the replication of severe fever with thrombocytopenia syndrome virus (SFTSV), a negative-sense RNA virus belonging to the *Bunyaviridae* family. The study further demonstrated that the SFTSV RNA-dependent RNA polymerase exhibits affinity for a specific SM lipid species and subsequently mapped the corresponding lipid-binding pocket within the polymerase structure [121]. Such mechanistic insights could ultimately guide the development of novel direct-acting antiviral inhibitors that target lipid-binding pockets and disrupt critical protein–lipid interactions. However, within the CoV field, the pace of mechanistic characterization has not kept up with the growing number of lipid metabolism inhibitors reported to exhibit antiviral activity. While many compounds have demonstrated promising antiviral effects, the underlying mechanisms responsible for these phenotypes often remain poorly defined. Addressing this knowledge gap will be essential for identifying the most relevant host targets, understanding the molecular basis of antiviral activity and enabling the rational development and optimization of future antiviral strategies.

Continued efforts to develop compounds targeting host pathways co-opted by multiple virus families may ultimately yield truly broad-spectrum antivirals. In principle, such inhibitors could be effective against viruses that depend on host membranes for the generation of their ROs (e.g. picornaviruses), the acquisition of a lipid envelope (e.g. influenza- and retroviruses) or both (e.g. flaviviruses and CoVs). The growing threat posed by emerging and neglected viruses, including other enveloped viruses such as flavi-, paramyxo-, filo- and bunyaviruses, underscores the urgent need for innovative therapeutic strategies that target conserved host processes rather than virus-specific factors. Despite the challenges regarding efficacy, toxicity and mechanistic understanding outlined above, host-directed modulation of lipid metabolic pathways remains a promising avenue for the development of next-generation antiviral therapies with the potential to provide protection against a broad spectrum of current and future viral threats.

## Abbreviations

2-BP: 2-bromopalmitate
ACC: acetyl-CoA carboxylase
ACSL: long-chain fatty acyl-CoA synthetase
AGPAT: acylglycerolphosphate acyltransferase
aSMases: acid SMases
ATGL: adipose triglyceride lipase
CoV: coronavirus
COVID-19: coronavirus disease 2019
DGAT: diacylglycerol O-acyltransferase
DMVs: double-membrane vesicles
DNL: *de novo* lipogenesis
ER: endoplasmic reticulum
FASN: fatty acid synthase
gRNA: RNA genome
GWAS: genome-wide association study
HCV: hepatitis C virus
HDL: high-density lipoprotein
MERS-CoV: Middle East respiratory syndrome coronavirus
nSMases: neutral SMases
nsps: nonstructural proteins
PA: phosphatidic acid
RAR*α*: retinoic acid receptor alpha
RO: replication organelle
SCAP: SREBP cleavage-activating protein
SFTSV: severe fever with thrombocytopenia syndrome virus
SM: sphingomyelin
SMases: sphingomyelinases
SR-B1: scavenger receptor class B type 1
SRE: sterol regulatory element
SREBP: sterol regulatory element-binding protein
TAG: triacylglycerol
TOFA: 5-(tetradecyloxy)-2-furoic acid
zDHHC: zinc finger DHHC-type
